# Biomimetic robots reveal flexible adjustment of sexual signalling in a wild invertebrate

**DOI:** 10.1098/rspb.2025.1570

**Published:** 2025-08-06

**Authors:** Joe A. Wilde, Safi K. Darden, Jordan D. A. Hart, Michael N. Weiss, Samuel Ellis, Tim W. Fawcett

**Affiliations:** ^1^ Centre for Research in Animal Behaviour, University of Exeter, Exeter EX4 4QG, UK; ^2^ Ecology and Environmental Science, Biomathematics and Statistics Scotland, Aberdeen AB15 8QH, UK; ^3^ Center for Whale Research, Friday Harbor, WA 98250, USA

**Keywords:** biorobotics, sexual signalling, courtship, sexual selection, tactical adjustment, fiddler crab

## Abstract

Studies of communication in primates, birds and fish show that signalling behaviour is often flexibly adjusted depending on the presence, characteristics and behavioural states of others. Such flexibility is likely important in competitive social contexts such as sexual signalling, where attractiveness is directly affected by rival behaviour. Although sexual displays are often sensitive to the presence and proximity of rivals, less is known about the effect of changes in rival signalling. In this study, we used a biomimetic robot to manipulate rival morphology and waving behaviour in a wild population of fiddler crabs (*Afruca tangeri*) and investigated whether males responded flexibly by adjusting their own activity and waving. Males were less likely to enter their burrow when the robotic rival was waving, particularly if that rival had a small claw, and spent less time in their burrow if they did enter. While outside, males produced more waves when the robotic rival was waving fast, not by changing their own rate but by prolonging their bouts. These results reveal the subtle ways in which an invertebrate flexibly adjusts behaviour to remain competitive in a dynamic environment, investing more in signalling when it is likely most profitable.

## 1. Introduction

Signalling in a range of non-human animals is often highly flexible, in that its expression is adjusted depending on the presence [1], composition [2] and even behavioural states [3] of the receivers. These receivers can encompass intended recipients as well as passive bystanders and potential eavesdroppers [4]. Examples of flexible signalling include various forms of gestural communication in non-human primates [4,5], birds [6,7] and fish [8], as well as vocal communication in non-human primates [2,9,10], birds [11–13] and anurans [14]. In chimpanzees (*Pan troglodytes*), for instance, the targets of attacks modify the acoustic structure of their screams to exaggerate the threat only if at least one bystander matches or exceeds the dominance rank of their attacker [2]. In fowl (*Gallus gallus*), subordinate males performing ‘tidbitting’ to advertise food discovery to nearby hens are less likely to produce audible vocalizations alongside their visual motor display if the alpha male is attentive (head up and not vocalizing), which reduces the speed and severity of the alpha male’s attacks [11]. In these examples, the signaller flexibly adjusts its behaviour in a way that seems adaptive, by enhancing the potential benefits of signalling while reducing the potential costs.

Mating competition is one important area in which there is likely to be strong selection for flexibility in signal expression. The effectiveness of sexual displays varies depending on the presence and receptiveness of potential mates, as well as competition from rival signallers. The cost of displaying scales with the threat from interceptive eavesdropping by predators, parasites and potentially aggressive rivals [15]. The ‘tactical adjustment’ hypothesis [15] proposes that signallers have been selected to modify their behaviour flexibly over short periods of time, in response to internal or external cues, to reduce costs and maximize the efficacy of their displays (note that, following other research in this area [16–20], this hypothesis is framed at the behavioural level, with nothing implied about the cognitive or non-cognitive mechanisms involved). Studies on diverse taxa have shown that males tactically adjust their sexual display behaviour in response to predator cues [21] and key aspects of their social environment, such as the distance to females [16,22], the level of female interest [16,18] and the number of displaying rivals [20,23]. However, although effects of rival presence and density have been found in both vertebrate [12,14] and invertebrate [20,23] animals, less is known about tactical adjustment of signalling in response to changes in rival behaviour.

Given that signallers often directly compete for a limited number of mating opportunities, we might expect individuals to adjust their own signalling behaviour in response to short-term changes in the signalling behaviour of nearby rivals, and for those rivals to do the same [15]. For example, if potential mates prefer signallers displaying at high rates and one signaller increases the rate at which they are signalling, this changes the relative attractiveness of all nearby competitors, which may prompt tactical adjustments. Disentangling these inter-dependent responses requires careful experimental manipulation of signalling behaviour, for which biomimetic robotics are an increasingly powerful tool [24–27]. In this study, we investigated tactical adjustment of sexual signalling behaviour in a wild population of the fiddler crab *Afruca tangeri*. We used a custom-built biomimetic robot to investigate experimentally how male crabs adjust their sexual signalling in response to fine-scale changes in the signalling behaviour of a rival male. We independently manipulated multiple aspects of the robot’s display and recorded how real *A. tangeri* males changed their own behaviour in response.

During the breeding season, male *A. tangeri* defend subterranean burrows (approx. 31−32 cm deep [28]) in coastal mudflats and sandbanks (up to 17 burrows per m^2^ [28], with an average density of 6 per m^2^ (J.A.W., 2022, unpublished data). The burrows are used for mating, but also as refuges for crabs to escape from predators and conspecific aggression, thermoregulate and re-hydrate [28–32]. During low tide in the breeding season, the crabs emerge on the surface to maintain their burrow, forage and either perform waving displays to attract mates (males) or search for and evaluate potential mates (females) [28].

Male *A. tangeri* have an enlarged chela (major claw) that they wave repeatedly to attract females [29]. Females have been shown to prefer males with a larger claw [33] and a faster waving rate [34,35]. Like repetitive displays in many other species [16,36,37], waving is performed in bouts: waves (raising and lowering of the claw, lasting 1 s or less) are interspersed with pauses that may be very brief (sometimes less than 1 s, within a signalling bout) or much longer (between signalling bouts) [28]. Males may alternate between periods of high-intensity waving (high speed, short inter-wave interval, high claw elevation) and low-intensity waving (slower speed, longer inter-wave interval, lower claw elevation). These two types of waving are employed differently across social contexts: high-intensity waving is used as a courtship signal to females, whereas low-intensity waving is used as a broadcast signal to attract females to the local area [22]. Because male *A. tangeri* cannot see or visually signal to females when they are underground, they directly trade off time spent signalling with time spent in their burrows.

The major claw can account for over 30% of a male’s total body mass [38]. This weight imposes high costs (high power input, *sensu* [39]) during locomotion [40], and waving his claw to court females reduces a male’s sprint speed (a predator-evasion behaviour) 1−2 h later [41]. It seems likely that waving the claw at a faster rate requires greater power input, and waving for longer periods entails a greater overall energetic (joule) cost [39]. Selection should therefore favour males that wave less when the expected benefits are lower. Consistent with this, males invest less in waving to smaller, less fecund females [42], and when a female is far away and the chances of mating are lower [22].

Using the biomimetic robot, we independently manipulated static (claw size) and dynamic (wave speed) aspects of a rival’s display and investigated effects on real males’ waving behaviour and their burrow use. Because males in their burrows cannot see any mate-searching females and cannot wave, for our purposes we consider the time a male spends in his burrow as an aspect of his sexual signalling strategy. We predicted, first, that focal males would spend less time in their burrow when the robotic rival was waving. Such a response might be expected to occur if males eavesdrop on rival male signalling to infer the location of mate-searching females; if waving by neighbouring rivals indicates that females are nearby, males should reduce the time spent in their burrow. Such eavesdropping has been demonstrated in a variety of species including wolf spiders (*Schizocosa ocreata* [43]), guppies (*Poecilia reticulata* [44]), whitethroats (*Sylvia communis* [45]) and another fiddler crab species (*Austruca mjoebergi* [46]). Our second prediction, based on the documented preference for faster-waving males [35], was that males would display at a faster rate when the robotic rival was waving, and even more so when it was waving fast. Third, we predicted that males would extend the duration of their display bouts when the robotic rival was waving, to remain competitive when assessed by females. Finally, given that females prefer males with larger claws [35], we also predicted that the claw size of the rival male would influence the courtship behaviour of focal males, as suggested by previous literature [33], because small-clawed and large-clawed rivals may present different levels of competition for mates.

## 2. Methods

### (a) Study area and species

The study was carried out between May and July 2022 on a wild population of *A. tangeri* that inhabit the Ria Formosa Natural Park on the south-eastern coast of Portugal, between Manta Rota and Cacela Velha (37.16N, 7.53W). At low tide, this area consists mainly of mudflats, salt marshes and dry sandy banks.

### (b) Robotic stimulus male

**Figure 1.**
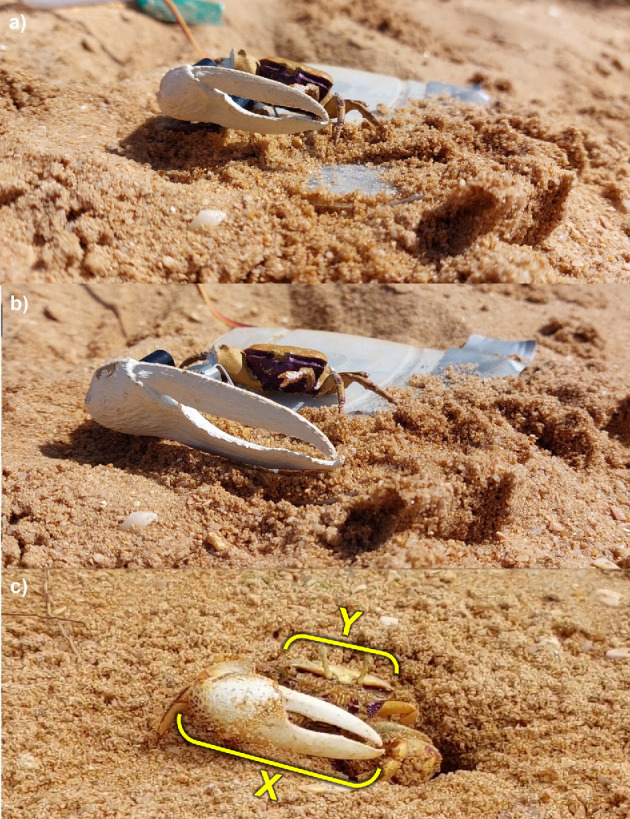
Biomimetic robotic rival male *A. tangeri* with the (a) small and (b) large claw attached, and (c) a real male *Afruca tangeri*. The yellow brackets indicate where the male’s claw length (*X*) and carapace width (*Y*) were measured. (*Photograph: Joe A. Wilde*)

To provide a standardized rival stimulus of different sizes, we used a three-dimensional (3D) scan of a fiddler crab specimen from a museum collection [47]. The body and major claw of this scan were 3D-printed separately by FabLab Devon (fablabdevon.org). The major claws were printed in two sizes: one where the major claw was 5 cm from manus base to pollex tip (the approximate mean length of male *A. tangeri* major claws in our records; see electronic supplementary materials) and one where the major claw measured 7 cm (the largest major claw length in our records; see electronic supplementary materials). The body was 3D-printed in one size: the size of the original scan when the major claw was scaled to the mean length (25.8 mm carapace width). After printing, the models were painted to closely resemble male *A. tangeri* colours to the human eye using acrylic paint (figure 1).

The printed major claw was glued to a mobile joint that allowed vertical movement. This joint was then fixed to a plastic base and a length of metal wire passed through the plastic base, allowing the printed claw to be joined to a Tower Pro Micro Servo SG90. This micro servo was attached to an Arduino Uno Rev3 microcontroller that controlled the timing of the micro servo movement and therefore the vertical movement of the printed claw, emulating the waving of a male *A. tangeri*. The Arduino microcontroller was also connected to an HC−05 Bluetooth serial transceiver, allowing the wave speed of the robot to be changed remotely via Bluetooth connection to a phone. The robotic stimulus male could wave at two speeds: a ‘slow’ speed of one wave every 2 s, simulating low-intensity waving; and a ‘fast’ speed of one wave every 1 s, simulating high-intensity waving (see electronic supplementary materials, video). These wave speeds were estimated from videos of real males collected in previous field seasons (S.K.D., 2010, unpublished data).

We assume that focal male behaviour reflects responses to any rival male rather than being specific to the scanned specimen that formed the basis of our 3D-printed models. We observed multiple male *A. tangeri* aggressively challenging and physically attacking the robotic rival during test trials (see electronic supplementary materials). Given that we have only observed *A. tangeri* fighting with conspecifics, we are confident that the focal males perceived the robotic rival as a conspecific rival male and our assumption of generalizability is justified.

### (c) Experimental set-up

All experimental trials were carried out during diurnal low-tide periods, between 8:00 and 17:00. Before the trial began, a male that was actively waving on the surface next to his burrow was selected as the focal male. All burrows within a 60 cm radius of the focal male’s burrow were plugged for the duration of the trial (approx. 20 min) using bundles of locally collected samphire (*Salicornia europaea*). This prevented any crabs that occupied nearby burrows from emerging onto the surface during a trial and temporarily removed all but the focal male from the experimental area. Crabs are adapted to spending 4−6 h in their burrow every high tide, so it is unlikely that the trapped individuals experienced any negative consequences, except for a 20-min period when food availability was restricted. Other males could still walk through the experimental area, and this was recorded when it happened. The robotic stimulus male was placed on the substrate 30 cm away from the focal male’s burrow entrance (approximately the average distance to the nearest neighbouring burrow; J.A.W., 2022, unpublished data) with either the ‘small’ or ‘large’ claw attached. Each focal male was only presented with one claw size (randomized across males) for the duration of their trial. Two GoPro Hero 4 cameras were then set up to record the behaviour of the focal male, one behind the robotic stimulus and one on the other side of the focal male’s burrow, facing the robotic stimulus. This camera set-up ensured that the frontal view of the focal male was always visible.

Once all equipment was set up, the experimenter (J.A.W.) retreated to a distance at which his presence would not affect focal male behaviour (*>*10 m) and a timer was started. If the focal male did not emerge from his burrow within 7.5 min, that trial was abandoned. If the focal male did emerge, the timer was reset to mark the beginning of the first condition of the trial and we moved through a sequence of four conditions with that male, each condition lasting for 5 min: (i) the robotic stimulus was present but not waving (‘No wave’ treatment); (ii) the robotic stimulus started waving at one of the two wave speeds (either ‘Slow wave’ or ‘Fast wave’ treatment); (iii) the robot waving was stopped and the focal male experienced another ‘No wave’ condition; and (iv) the robot waved at the speed that the focal male had not experienced in condition ii. All changes to robot wave speed were made remotely to avoid disturbing the focal male.

The time at which the trial started was noted, as well as the substrate surface temperature (measured using a Magnusson KC-180A1 infrared thermometer, ±2°C). The claw size treatment and order of wave speed presentation were balanced across focal males using a randomized-blocks design [28]: each ‘block’ consisted of four trials covering the four possible experimental conditions (large, slow→fast; small, slow→fast; large, fast→slow; small, fast→slow), with these applied to four different males in fully randomized order. The same ‘small’ and ‘large’ robot claws were used across all focal males in each claw size treatment and the same robot body was used for all focal males.

### (d) Focal male morphometrics

Once a trial had ended, all apparatus was removed from the area and a tube trap [28] was placed in the entrance to the focal male’s burrow. If the focal male entered the trap (27 males out of 58 used for the final analysis), we measured the distance between the base of his manus and the tip of his pollex (claw length) and the widest point of the dorsal side of his carapace (carapace width; figure 1) using a Whitworth electronic calliper (±0.01 mm), and weighed him using Ohaus Traveller’s scales (±0.1 g). The captured males were then marked with a non-toxic paint pen on the carapace to avoid using the same male twice across trials.

If the focal male did not enter the trap within 20 min (31 males out of 58 used for the final analysis), the trap was removed and his claw length and carapace size were estimated from the GoPro footage using the software ImageJ [48], with a measuring tape placed above his burrow in the video footage for scale. Because these males were not caught, they could not be marked to avoid reusing them. However, their burrows were avoided when selecting males for future trials. Given that individuals of this species occupy their burrows for 1−2 weeks [28] and given the large population (many thousands of individuals) occupying the mud flats in the study area, the chances of accidentally using the same male twice for a trial is very low.

The experimental trials were carried out on 70 focal males, but only 58 were used for the final analysis because some trials were either interrupted by human passers-by (5 trials) or the focal male left the experimental area during the trial, usually after losing a fight with a wandering male (7 trials). Table 1 shows the focal male and trial information for each of the four experimental treatments, as well as information on the trials not included in the final analysis.

**Table 1.** Information about the trials in each of the four experimental conditions, as well as the trials not included in the analyses owing to interruptions. Claw length and carapace width refer to the focal male.

robot claw size	presentation order	number of trials	mean claw length (mm)	claw length S.D. (mm)	mean carapace width (mm)	carapace width S.D. (mm)	mean temperature (° C)	temperature S.D. (° C)	trials interrupted by passers-by	trials in which male left area before end of trial
large claw	fast→slow	14	44.4	11.2	25.9	4.1	24.3	3.6	0	3
large claw	slow→fast	13	45.7	9.5	26.8	4.8	24.6	4.2	3	1
small claw	fast→slow	18	41.9	12.5	26.4	4.8	23.7	3.4	0	2
small claw	slow→fast	14	43.6	10.6	27.2	4.2	24.3	3.3	2	1

### (e) Behaviour coding and data

For each experimental trial, we extracted all time points at which the focal male entered or exited his burrow, or performed a wave with his major claw (the definitions for these behaviours are in table 2). We then calculated the proportion of time that the focal male spent in his burrow during each wave speed treatment. We also recorded the number of males in frame that were not the focal male, as well as the number of females in frame (i.e. within a maximum of 60 cm of the focal male), at each point in the videos.

**Table 2.** Ethogram showing detailed definitions of the behaviours coded and used in subsequent analyses. Point events are one-off occurrences of a behaviour over a short time frame (maximum 1–2 s), while state events are a change in state that lasts for a longer time frame (2 or more seconds) [49].

Behaviour	Type	Description
aggressive stance	point event	male opens and raises claw in aggressive stance. (Not waving)
wave	point event	male waving enlarged claw above the level of their eye and returns claw to resting position.
in burrow	state event	all of male's legs are not visible in camera footage.
out of burrow	state event	all of male's legs are visible in camera footage and the male is not engaging in any other behaviours.
fight	state event	male makes physical contact with another individual.
female is visible in camera footage	point event	female enters frame.
female exits camera footage	point event	female leaves frame.

Videos were coded once by one of the research team (J.A.W.) who was not blind to the claw size or wave speed treatment. A second person who was unaware of the aims of the study independently coded 10% of the trial videos in which the robotic rival was not visible; this person was therefore blind to both the claw size and wave speed treatments. All behaviour coding was carried out using BORIS [49]. The inter-rater correlation coefficient (also known as the intra-class correlation coefficient, ICC; calculated in R using Stan and Cmdstan [50–53]) for the total number of waves was 0.87 (89% highest density interval (HDI) [0.76, 0.97]), and the ICC for the time spent not waving was 0.89 (89% HDI [0.81, 0.98]). The ICC for time spent in burrow was 0.87 (89% HDI [0.77, 0.97]). These values indicate ‘good’ to ‘excellent’ reliability for our behavioural measures [54].

### (f) Statistical analyses

#### (i) Burrow use

We used a linear hurdle model to analyse whether males adjusted the time spent in their burrow in response to changes in the behaviour and claw size of the robotic rival. In this analysis, we asked how the signalling behaviour of the biomimetic rival male affected (i) the probability that a male entered his burrow and (ii) how long he spent there. For each of the 5-min wave speed conditions (‘No wave’, ‘Slow wave’ or ‘Fast wave’), the probability that the male entered his burrow at all was modelled using the Bernoulli distribution (binary outcome, the ‘hurdle’). For those cases where the male did enter his burrow, the proportion of the 5 min that he spent there was modelled using the Beta distribution (ranging between 0 and 1). In both parts of the linear hurdle model, the experimentally manipulated predictorsthat we included were the wave speed of the robot and the claw size of the robot, as well as the interaction between the two. We also included treatment order and the following uncontrolled predictor variables: the focal male’s carapace width; his claw-length-to-carapace-width ratio; the substrate surface temperature at the start of the trial; and the time when the trial started relative to the lowest tide point. Previous studies have found that fiddler crab behaviour is statistically associated with each of these variables [28], so we included them as fixed effects to refine our tests of the experimental treatment effects. Occasional visits from females and rival males were ignored in these models as they happened on a much quicker timescale than the response variable, which is a summary of a 5-min period. Random intercepts were included for focal male identity to account for non-independence among measurements and to capture natural variation among males in their overall tendency to enter and spend time in the burrow.

#### (ii) Signalling behaviour

To analyse the male’s behaviour when he was outside his burrow, the data were divided into 5-s time intervals. Any 5-s intervals during which the focal male left the field of view of both video cameras at any point were discarded. In each of the remaining intervals, we recorded the number of times the focal male waved and the number of females in frame, as well as the number of wandering rival males (in view of either of the two GoPro cameras). This gave a total of 10 400 5-s intervals across 58 individuals. We chose 5 s as the time interval for analysis to balance the amount of detail we retain in behavioural sequences (which is higher when the data are divided into small intervals) against the computational processing time required to analyse the data (which is also higher when the data are divided into many small intervals).

**Figure 2.**
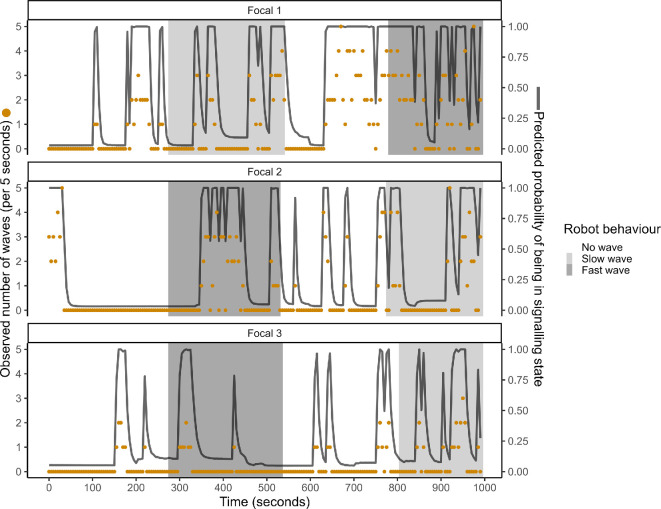
The observed number of waves (orange dots) by a focal male every 5 s over 20 min in three different example trials, and the estimated probability (grey line) from a hidden Markov model that the focal male was in the signalling state. The greyed areas (which indicate the robot behaviour conditions) do not perfectly align because the timings of each treatment varied slightly owing to logistical constraints in the field.

We analysed the resulting data using hidden Markov models (HMMs) [16,55,56], a data-driven approach that identifies the bout structure of repetitive sexual displays such as claw waving, avoiding the pitfalls of researcher-defined bout criteria that have been used in the past (discussed in [16]). We modelled focal males as being in one of two ‘hidden’ (underlying) states: a signalling state (actively displaying) and a non-signalling state (visible above ground but not actively displaying). We used the number of waves per 5 s as a proxy from which to infer the underlying state of the male. By fitting HMMs to the full sequence of 5 s time intervals in a trial, we could simultaneously estimate the male’s waving rate in the signalling state and his probability of transitioning between states (from signalling to non-signalling, or *vice versa*) from one interval to the next, capturing the temporal autocorrelation between behaviours in successive intervals. Figure 2 is a visual representation of how these models work.

The HMMs also allow us to estimate how the probabilities of transitioning between states and waving rates depend on predictor variables characterizing the male and his social context. We estimated the effect that each predictor variable has on (i) the probability that a focal male stays in the signalling state, (ii) the probability that he stays in the non-signalling state, (iii) the rate of waving when in the signalling state and (iv) the (low) rate of waving when in the non-signalling state. The experimentally manipulated predictors that we included were the wave speed of the robot, the claw size of the robot and their interaction. As before, to control for additional sources of variation and refine our tests of the experimental treatment effects, we also included the following uncontrolled predictor variables known to be associated with fiddler crab behaviour [22,28,57,58]: the number of females in view (of either GoPro) in each 5-s interval; whether a wandering rival male was in view in each 5-s interval; the substrate surface temperature at the start of the trial; the focal male’s carapace width; his claw-length-to-carapace-width ratio; and the time of the 5-s interval relative to the lowest tide point. Random intercepts were included for focal male identity to account for non-independence among measurements and to capture natural variation among males in their average transition probabilities and waving rates. A full outline of the models used can be found in equations (1) and (2) in the electronic supplementary materials.

#### (iii) Model fitting

All models were written in Stan [51] and compiled and run using CmdStanR v. 0.5.3 [50] and CmdStan v. 2.30.1 [51] in R v. 4.1.2 [52,53] on the UK Crop Diversity high-performance computing platform [59]. Weakly informative priors were used for all parameters and checked using prior predictive checks (full model information including priors in electronic supplementary materials). Model convergence was checked using trace plots, posterior predictive checks and the potential scale reduction factor ($\hat{R}$) [60]. To increase stability of the reported intervals, and following convention laid out by [61], the 89% HDI of effect sizes (unless otherwise stated) is reported throughout [61,62]. We also report the proportion of posterior samples indicating an effect in the opposite direction from the reported effect, denoted either as Pr(*β* > 0) if the majority of the posterior density mass is negative, or as Pr(*β* < 0) if the majority of the posterior density mass is positive; thus small values of this proportion suggest that the reported effect is clearly distinguishable from zero. All continuous predictors (temperature, time, carapace width, claw : carapace ratio) were converted to *z*-scores prior to analysis to facilitate model convergence and aid interpretation. All effect sizes (*β*) are reported on the link scale. Therefore, effects on transition probabilities represent the change in log-odds associated with a 1-unit increase in the predictor. Effects on wave rates represent the change in the logarithm of the wave rate associated with a 1-unit change in the predictor. Because we used a Bayesian mixed-modelling framework with weakly informative, regularizing priors, the risk of type I errors in these models is lower than in other frameworks [63]. A simplified version of this analysis was run without non-experimental predictors to ensure robustness of the full model. This simplified model found the same results and is outlined in electronic supplementary material, table S8.

## 3. Results

The summary of all model outputs, including prior distributions, posterior means, s.e and effective sample sizes for all parameters, can be found in the electronic supplementary material. $\hat{R}$ ≈ 1 for all parameters for both models [63].

Overall, males performed an average (median) of 12 (interquartile range (IQR) = 35) waves throughout the ‘slow wave’ condition and 13 (IQR = 32.3) throughout the ‘fast wave’ condition. During the first ‘no wave’ condition, males performed an average of 6.5 (IQR = 31.5) waves, and 14 (IQR = 44) throughout the second ‘no wave’ condition. These differences could potentially result from changes in burrow use, signalling behaviour or both, as we explore below.

### (a) Burrow use

**Figure 3.**
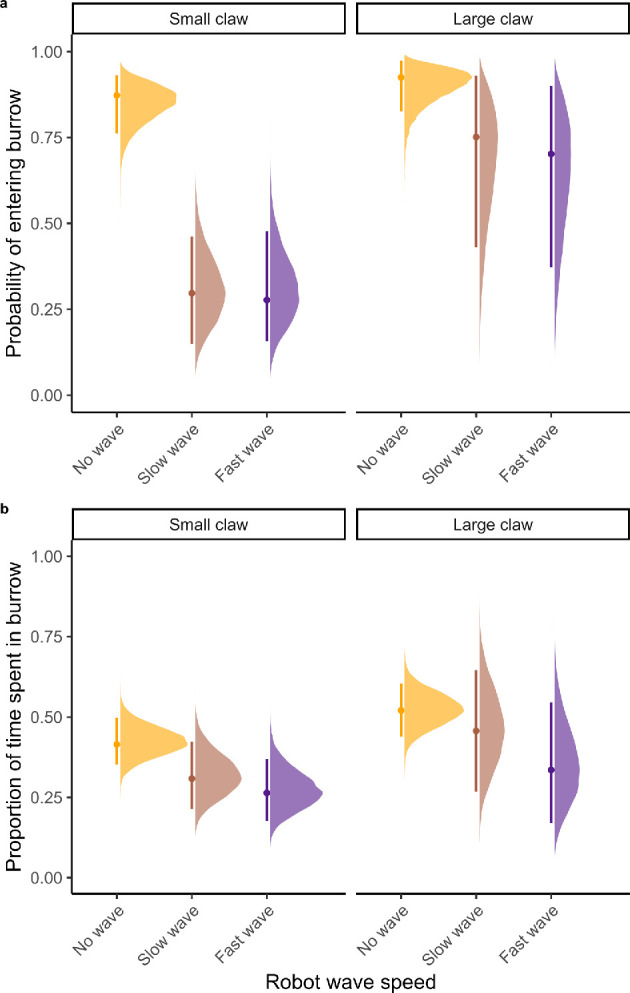
Posterior predictions from the linear hurdle model of (*a*) the probability that a male entered his burrow and (*b*) the proportion of time he spent in his burrow for each robot wave speed and robot claw size. The points show the posterior mode, and the lines span the 89% highest density interval for each wave speed condition.

There was evidence of an interaction between the effects of robot claw size and robot waving on the probability that males entered their burrow (figure 3). Males were most likely to enter their burrow when the robot was not waving, and claw size had no effect on this (no wave × large claw effect size 89% HDI [−0.39, 1.35], Pr(*β* < 0) = 0.17). However, when the robot was waving either ‘slow’ or ‘fast’, males were less likely to enter their burrow if the robot had a small claw (slow wave × small claw [−3.46, −1.82], Pr(*β* > 0)<0.001; fast wave × small claw [−3.38, −1.77], Pr(*β* > 0) < 0.001) than if it had a large claw (slow wave × large claw [−2.02, 0.07], Pr(*β* > 0) = 0.07; fast wave × large claw [−2.22, −0.12], Pr(*β* > 0) = 0.04). No other predictors showed any effects on the probability of entering the burrow (see model in electronic supplementary material, table S4).

If males did enter their burrow at all, they spent less time in their burrow when the robot was waving versus not waving, either ‘slow’ ([−0.92, 0.03], Pr(*β* > 0) = 0.06) or ‘fast’ ([−1.16, −0.20], Pr(*β* > 0) = 0.01; *β*_Fast_ – *β*_Slow_: [−0.85, 0.38]). When the robot had a large claw, males spent more time in their burrow than when the robot had a small claw ([0.02, 0.78], Pr(*β* < 0) = 0.05). However, there was no evidence of an interaction between the effects of robot wave speed and robot claw size on time spent in the burrow (slow wave × large claw [−0.52, 0.86], Pr(*β* > 0) = 0.36; fast wave × large claw [−1.07, 0.47], Pr(*β* > 0) = 0.26). Males also spent less time in their burrow at warmer temperatures ([−0.39, −0.05], Pr(*β* > 0) = 0.02) and less time in their burrow when the ‘fast’ wave speed condition was presented first ([−0.70, 0.00], Pr(*β* > 0) = 0.05). No other predictors had a clear effect (see model output in electronic supplementary material, table S4).

### (b) Signalling behaviour

We found no evidence of an interaction between the effects of robot wave speed and robot claw size on any of the parameters (electronic supplementary material, table S7), so these higher-order terms were removed from the model. There was a clear difference in wave rate between the two hidden states identified by the model: the estimated wave rate (intercept term posterior mean) was 0.02 ([0.01, 0.03]) waves per 5 s in the non-signalling state and 1.4 ([1.04, 1.86]) waves per 5 s in the signalling state (electronic supplementary material, figure S5). There is little evidence to suggest that the robot’s behaviour and claw size or any other predictors had an effect on the rate of waving in the signalling state. The 89% HDI of the predicted wave rate per 5 s for each condition of the robot rival were as follows: ‘no wave’ and ‘small claw’ (Intercept) [1.01, 1.82], ‘slow wave’ [0.97, 1.86], ‘fast wave’ [1.04, 1.88], ‘large claw’ [1.01, 1.81] (see predicted effect sizes in electronic supplementary material, table S5). Because the wave rate during non-signalling was close to zero and we had no prior expectation that any of the predictors would affect this, we provide full information on the estimated effects in the model output in the electronic supplementary material, table S5.

**Figure 4.**
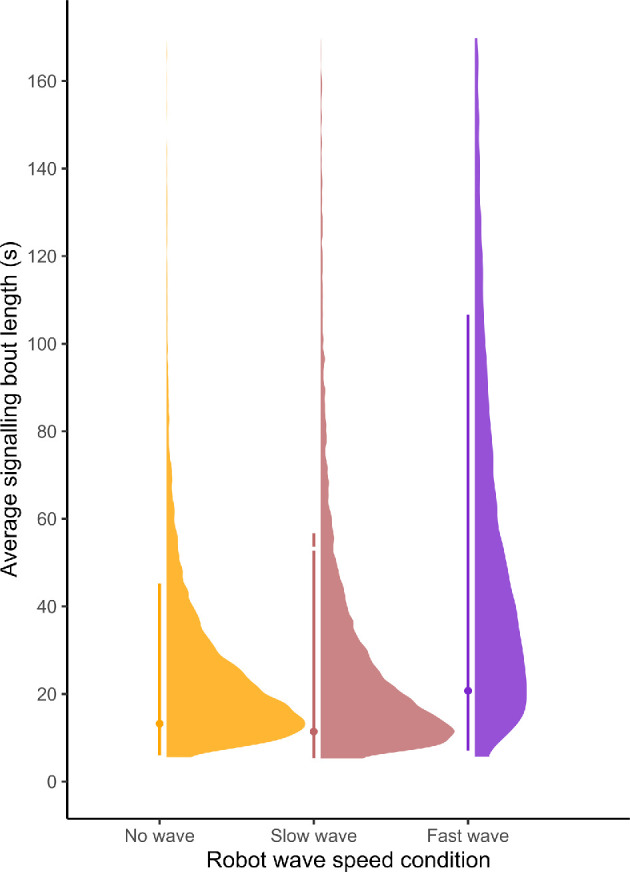
Posterior predictions from the hidden Markov model of the average length of signalling bouts for the three robot wave speed conditions. The points show the posterior mode and the lines span the 89% highest density interval for each wave speed condition.

Males had a higher probability of remaining in the signalling state when the robotic rival stimulus was waving at the ‘fast’ speed compared with when the robot was not waving ([−0.02, 2.03], Pr(*β* < 0) = 0.06), leading to longer signalling bouts in this treatment condition (figure 4). There was no apparent effect of the robot waving at the ‘slow’ speed ([−1.09, 1.04], Pr(*β* < 0) = 0.49) or the claw size of the robot ([−1.25, 1.27], Pr(*β* < 0) = 0.50) on the probability of remaining in the signalling state.

There is little evidence to suggest that any other predictors had an effect on the probability of remaining in a signalling bout (see model output in electronic supplementary material, tables). The behaviour and claw size of the robotic stimulus did not appear to affect the probability of remaining in a non-signalling bout (‘slow wave’ [−1.32, 0.81], ‘fast wave’ [−1.49, 1.19], ‘large claw’ [−1.29, 1.28]; Pr(*β* > 0): ‘slow wave’ = 0.35, ‘fast wave’ = 0.38, ‘large claw’ = 0.50); non-signalling bouts therefore lasted for similar times across all robot treatments. There is also little evidence to suggest that any other predictors affected the probability of remaining in a non-signalling state (see model output in electronic supplementary material table S6).

A summary of the effects of the robotic rival can be found in table 3.

**Table 3.** A summary of the effects that the robotic rival had on the behaviour of the focal males, showing the direction of the effect when following the arrow’s direction. The effects of all predictors included can be found in the full model output in electronic supplementary material, table S4–S6. Asterisks indicate where there is evidence of an interaction between waving and claw size.

	Signalling behaviour			Burrow use	
Experimental Condition	Wave rate	Signalling bout duration	Non-signalling bout duration	Prob. entering burrow	Prop. time spent in burrow
Claw size (small → large)	—	—	—	INCREASE${\;}^{*}$	INCREASE
No wave → Waving	—	INCREASE	—	DECREASE${\;}^{*}$	DECREASE
Wave speed (slow → fast)	—	INCREASE	—	—	DECREASE

## 4. Discussion

In this experimental study of wild fiddler crabs, we used a biomimetic robot to manipulate the static (claw size) and dynamic (wave rate) aspects of rival displays, and detected a multifaceted behavioural response in which males adjusted both the use of their burrow and their signalling behaviour. Male crabs were more likely to stay above ground when the robotic rival was waving compared with when it was present but not waving, and the response to waving was strongest if that rival had a small claw rather than a large claw. When males did enter their burrow, they spent less time in there when the robotic rival was waving rather than not waving, and when the ‘fast’ wave speed treatment was presented first, and if the rival had a small claw rather than a large claw. Outside their burrow, we found that males maintained a similar wave rate within display bouts regardless of the rival’s claw size and wave speed. However, males extended their display bouts for longer when the robotic rival was waving at a fast rate, compared with waving slowly or not at all. To our knowledge, this is the first evidence that an invertebrate animal adjusts its sexual signalling behaviour as a direct response to changes in the signalling behaviour of ostensible rivals.

By analysing an individual’s signalling strategy *in situ*, as we have done here, we can begin to disentangle some of the complex and sometimes antagonistic effects of the signaller’s social environment. Males were less likely to end a signalling bout when the rival was waving fast, meaning that the signalling bouts lasted longer during this treatment condition than when the rival was present but not waving, or when it was waving slowly. Because males typically use fast waves when females are nearby [22], it seems likely that the focal males were eavesdropping on the wave rate of the robotic rival and using this as a cue to the presence of nearby females. Males eavesdropping on the signalling of rival males to gain information about females has been documented in guppies [44], wolf spiders [43], whitethroats [45] and an Australian species of fiddler crab (*Austruca mjoebergi*) [46]. By using the behaviour of rival conspecifics to effectively extend the distance at which mate-searching females can be detected, males can tactically adjust their behaviour to increase signalling effort only when it is most effective in attracting females.

Similarly, the observed reduction in burrow usage when the rival was waving (compared with not waving) may be owing to rival signalling potentially indicating female proximity; it is in the best interests of a male not to enter his burrow when there is an immediate opportunity to attract a female. This explanation fits with the effect that we found of treatment order, whereby males spent less time underground when they experienced the ‘fast wave’ condition first (compared with experiencing the ‘slow wave’ condition first). If males use the fast waving of rivals as an indication that a female is nearby, they may benefit from remaining above ground for some time after that waving has ceased in order to avoid missing any mate-searching females that may still be wandering through the area. We also found effects of the rival’s claw size on burrow usage: if the robot had a small claw rather than a large claw, males spent less time in their burrow overall and showed a steeper drop in the probability of entering their burrow when that robot was waving. This may be an adaptive response to the fact that focal males are less likely to outcompete a large-clawed rival for a female’s attention [33], making time outside their burrow less profitable compared with when their rivals are small-clawed. Another potential explanation is that this is a strategy to avoid aggression from stronger conspecifics. If focal males perceived the large-clawed robot as a threat then their increased likelihood of entering their burrow and greater reluctance to emerge again may have been a defensive strategy to avoid attack and prevent their burrow from being stolen [64]. There is some evidence to suggest that males use rival claw size to assess fighting ability ([65]; but see [66]). Further experiments manipulating female presence as well as rival behaviour would be needed to distinguish between these explanations.

Given that there was no detectable effect of the wave speed treatment or of the robot’s claw size on the focal male’s wave rate, we did not find evidence that *A. tangeri* males tactically adjust their waving effort within bouts in response to changes in their relative attractiveness caused by changes in rival signalling. This finding fits with previous work showing that male *A. tangeri* do not change their wave rate in response to video playback of a male waving at mostly slow speeds [58], and that males of another fiddler crab species (*Austruca annulipes*) do not change their wave rate in response to 1−2 waving rivals [23]. However, changes to a male’s relative display efficacy are likely to be greater when competing with more males, so it remains possible that such adjustments in waving rate might be seen in response to signalling from a large number of rivals. Formal models are needed to identify evolutionarily stable strategies for signalling in such competitive situations.

The use of HMMs allowed us to infer the underlying state of the focal male (signalling versus non-signalling) and any adjustments that he made to the bout structure of his displays. These models worked well in estimating state (figure 2) based on the sequence of wave rates per 5-s interval for each individual, and identified clear differences between the wave rates within each state (see figure 2). HMMs are being used more frequently in ecology [55,56] and are a powerful tool for analysing the underlying state dynamics of many systems. However, they do require a sizeable amount of clean data to detect subtle effects or the effects of events that are sparsely represented in the data. For example, we found no strong evidence that male signalling was directly affected by females wandering through the experimental area. Studies on fiddler crabs have shown that males do react to females in the wild by waving at a higher intensity [29,35,58,64] and we have observed this phenomenon in *A. tangeri* at this field site. Therefore, we expected to see an effect of female presence in our data as we know this effect exists. However, females were present in the trial area for only 4.7% of the 5 s periods. It is possible that we would have been able to detect an effect of female proximity if they had appeared more often in the sample, but this was not the focus of our experimental study. Nonetheless, we were able to control statistically for any effect of female presence while investigating the effects of the robotic rival on signalling state and structure.

There are many examples of flexible signalling in vertebrate animals, including various forms of gestural communication [3,5,6,8], that seem much more complex than the waving displays of fiddler crabs investigated here. In some non-human primates [3,5], birds [6] and fish [8], studies have shown that signallers are sensitive to the recipient’s attentional state (e.g. their gaze direction), which some consider to be a hallmark of first-order intentionality [67,68]. Here, we have shown a simpler form of flexible communication in a small invertebrate animal, without requiring or implying anything about the physiological or cognitive mechanisms underlying this flexibility. Alongside other invertebrate studies showing sensitivity to recipient behaviour [12,14], our findings suggest that such flexibility may be more widespread in animal communication than previously thought.

In summary, our study shows that male fiddler crabs reduce burrow use and prolong their signalling bouts in response to high-intensity waving from a nearby rival, most likely because rival signalling is used as a cue to nearby females. However, we found no evidence that males tactically adjust their signalling intensity in response to changes in their relative attractiveness caused by rival behaviour. By using a biomimetic robot to manipulate behaviour experimentally, our study highlights the complex interplay between male fiddler crabs’ sexual signals and their response to both static and dynamic characteristics of rival males. We hope that this work will not only inform future research on the proximate and ultimate explanations of signalling behaviour in fiddler crabs but also inspire broader investigations into the role of competition and flexibility in shaping animal communication systems across diverse taxa. Future research should integrate these findings into a broader theoretical evolutionary framework to understand what forms of tactical signalling adjustment we would expect to evolve, and under what conditions.

## Data Availability

The data that support the findings of this study can be found in Dryad [69]. Supplementary material is available online [70].
